# Supplementary material for: Exploring the Experiences and Perspectives of Division III Athletes Regarding Personalized Nutrition Plans for Improved Performance—A Qualitative Investigation
Source: Healthcare (Basel). 2024 Apr 30;12(9):923. doi: 10.3390/healthcare12090923 (PMC11083372; doi:10.3390/healthcare12090923)
Supplement: Supplementary file 1 [file healthcare-12-00923-s001.zip › healthcare-2969636-supplementary.pdf]

Table S1: Quotes Used to Make Themes

Theme 1: Nutritional Knowledge and Awareness - Participants demonstrated varying levels of understanding regarding personalized nutrition plans.	
**Participant**	**Specific Transcript Excerpt**
P8	"I thought I knew a lot… about nutrition, but now I realize there's so much more to learn."
P14	"I always thought carbs were bad, but my nutritionist explained why they're important for energy [sigh]."
P22	"Honestly, I never paid much attention to nutrition until I… started feeling sluggish during games [laugh]."
P5	"We used to think protein was the only thing that mattered, but now I understand the importance of balance."
P18	"I'm interested in nutrition, but… there's so much conflicting information out there, it's hard to know what's right."
P10	" [laugh] You had no idea hydration was so crucial until I experienced dehydration during a match."
P26	"I thought supplements were the key to performance, but my coach… emphasized the importance of real food."
P3	"We never realized how much food affects recovery until I started focusing on nutrition."
P19	"I used to think I could eat whatever… I wanted because I burned so many calories, but I learned the quality of food matters too [sigh]."
P12	"No one understood why I felt so… tired [laugh] until I learned about fueling properly before workouts."
P24	"I've always been interested in nutrition, but I didn't… realize how much it could impact my athletic performance."
P7	"You never knew what a macronutrient was until my nutritionist explained it to me in simple terms."

P15	"I thought supplements… were necessary for gains, but now I see the importance of getting nutrients from whole foods."
P20	"We used to think all fats were bad, but I learned… about healthy fats and their role in brain function."
P11	"I didn't understand why…. hydration was important until I experienced cramps during practice [sigh]."
P17	"[laugh] We all thought eating less would help me lose weight, but I learned about the importance of fueling my body properly."
P23	"I never paid attention to nutrition until I started feeling sluggish… during workouts."
P9	"No one realized how much food affects performance until I started paying attention to my pre-game meals."
P16	"You thought I was eating healthy, but…my nutritionist showed me where I could make improvements."
P4	"I never thought about nutrition until I hit a plateau in my training and realized I needed to change something."
P21	"I used to think supplements… were the answer to everything, but now I focus on whole foods for better results [sigh]."
P25	"We always thought nutrition was complicated, but my coach broke it down into simple terms that made sense."
P1	"I thought hydration was just about drinking water, but now I know electrolytes are important too."
P13	"It was easy to skip meals to lose weight, but now I… understand the importance of fueling my body throughout the day [laugh]."
P27	"I never paid attention to nutrition until I started working with a nutritionist who explained how it could improve my performance."

P6	"We all thought I was eating well, but my nutritionist helped me see where I was missing key nutrients."
P2	"[laugh]You never realized…. how much timing mattered until I learned about pre- and post-workout nutrition."
P28	"I used to think supplements were a shortcut, but now I understand the importance of a balanced diet [sigh]."
P30	"No one understood why I felt so fatigued until my coach explained…. the role of nutrition in recovery."
P29	"I always thought nutrition was complicated, but my nutritionist showed me… simple changes I could make for big improvements."

Theme 2: Perceived Benefits of Personalized Nutrition Plans - Athletes expressed positive outcomes such as improved performance and overall well-being.

**Participant**	**Specific Transcript Excerpt**
P14	"Since I started following my personalized nutrition plan, I've noticed a significant improvement in my energy levels during workouts."
P22	"My recovery has been much… better since I started paying more attention to what I eat."
P8	"[laugh]I used to struggle with fatigue, but ever since I adjusted my diet, I feel like I can push myself harder in training."
P17	"I feel like I'm performing at my best now that I have a… nutrition plan tailored to my needs."
P3	"My teammate saw a difference in… my performance on the field since I started fueling properly."
P25	"I used to feel drained after games, but now I have more endurance and can keep up with the pace."
P10	"I've seen a big difference in my recovery time since I started focusing on nutrition."
P28	"My coach…. always says, 'You can't out-train a bad diet,' and I've definitely seen the truth in that [sigh]."

P19	"I feel like I'm getting stronger and faster now that I'm fueling my body properly."
P9	"I used to struggle with cramps during matches, but since I started hydrating better and paying attention to my nutrition, they've pretty much disappeared."
P21	"We have all noticed a huge improvement in my…. mood and overall well-being since I started eating better [sigh]."
P16	"I used to hit a wall halfway through… workouts, but now I have more sustained energy."
P4	"I feel like I recover faster now, which means I can train harder and more often [laugh]."
P27	"I've noticed that I'm more alert and focused during practice and games since I started following my nutrition plan."
P18	"My whole family struggles with digestive issues, but since…. I changed my diet, they've pretty much gone away [laugh]."
P7	"I feel like I'm sleeping better now that I'm eating better, which has a big impact on my recovery."
P1	"I've seen a difference in…. my body composition since I started paying attention to my nutrition."
P20	"Feeling sluggish all the time, but now I have more energy to get through the day."
P12	"Now that I am fueling properly, I can push myself …harder."
P29	"I've noticed improvements in my endurance and stamina since I started focusing on my nutrition."
P15	"I used to feel bloated and uncomfortable… after meals, but now I feel satisfied and energized."
P23	"My recovery time has improved, so I'm ready to go for the next practice or game much sooner."

P5	"I feel like my immune system is… stronger now that I'm eating better, so I'm not getting sick as often."
P26	"I used to struggle with mood swings, but now I feel more stable and positive."
P6	"We've noticed that I recover faster from injuries since I started paying more attention to…. my nutrition."
P2	"I used to have trouble focusing during workouts, but now I'm more dialed in and present."
P24	"We feel like I have more energy throughout the day, not just during workouts."
P11	"I've noticed that I have fewer …aches and pains since I started eating bette [sigh]r."
P30	"You felt bloated and uncomfortable after meals, but now I feel satisfied and energized."

Theme 3: Challenges and Barriers to Implementation - Various obstacles, including time constraints and conflicting dietary advice, hindered plan adherence.

Participant	Specific Transcript Excerpt
P14	"I struggle to find time to meal prep with my busy schedule...sometimes it's just easier to grab fast food."
P22	"There's so much conflicting information out there about what's healthy and what's not...it's overwhelming."
P8	"I don't always have [laugh]… access to the foods I need, especially when I'm traveling for games."
P17	"My teammates don't always understand why I eat…. certain things, so there's pressure to conform."
P3	"I have a sweet tooth, so it's hard to resist junk food temptations, especially when everyone else is indulging."
P25	"Eating healthy can be …expensive, and as a college athlete, I'm on a tight budget."
P10	"Sometimes I'm just too tired to cook after practice, so I end up grabbing whatever's convenient."

P28	"I've been told conflicting things about what to eat to fuel my workouts, so I'm not sure who to trust."
P19	"I have to balance academics, athletics, and social life, so nutrition often takes a …backseat."
P9	"When I'm stressed, I tend to reach for comfort foods, even though I know they're not the best for me."
P21	"I don't always have time to…. sit down and eat a proper meal between classes and practice."
P16	"[laugh]My family… doesn't always buy the foods I need at home, so I have to make do with what's available."
P4	"I've had coaches who didn't prioritize nutrition, so it wasn't something I thought about until recently."
P27	"I'm surrounded by… teammates who eat junk food all the time, so it's hard to stick to my nutrition plan."
P18	"I'm lactose intolerant, so finding dairy-free options that still provide enough protein can be challenging."
P7	"I feel pressure to maintain a certain body image, which sometimes conflicts with what's best for my performance [sigh]."
P1	"I struggle with portion control, especially when I'm stressed or… emotional."
P20	"My coach expects us to be at our best on the field [laugh] but doesn't provide much guidance on how to fuel properly."
P12	"I have to eat at the dining hall on campus, and the options are limited and not always the healthiest."
P29	"I'm not a fan of… cooking, so I often rely on pre-packaged meals or takeout, which aren't always the healthiest choices."
P15	"I have teammates who constantly talk about fad diets and quick fixes, which… can be distracting and misleading."

P23	"Sometimes I'm so focused on my training that I forget to eat, which leaves me feeling depleted and sluggish."
P5	"When I'm traveling for… away games, it's hard to find nutritious options on the road."
P26	"I often feel pressured to eat certain foods to fit in with my teammates, even if they don't align with my nutrition goals."

Theme 4: Influence of Team Culture and Environment – Team dynamics and cultural norms significantly influenced athletes' attitudes towards nutrition plans.

Participant	Specific Transcript Excerpt
P11	"Our team captain sets the tone for nutrition...if he's eating healthy, we all tend to follow suit."
P24	"There's a lot of pressure to… conform to the team's dietary norms, even if it's not what's best for me."
P6	"We have team dinners before big games, and it's usually pizza or burgers...it's like a tradition [sigh]."
P13	"When the coaches prioritize nutrition, it sends a message that it's important for our performance."
P2	"If the coaches talk about nutrition ….and fueling properly, it becomes part of the team culture."
P30	"Some of my teammates joke about eating junk food and skipping meals, but it's not really funny when it affects our performance."
P8	"Our team nutritionist provides us with guidance and meal plans, which helps reinforce the importance of nutrition."
P19	"The older players on the team influence the younger ones...if they're eating healthy, it sets a good example."
P17	"We have nutrition challenges…. and competitions within the team to encourage healthier eating habits."
P23	"We're all in this together, so we hold each other accountable for our nutrition choices."

P5	"Sometimes the team environment makes it hard to prioritize nutrition...it's easier to go along with what everyone else is doing [laugh]."
P22	"We have team meetings where we discuss nutrition and its impact on performance...it's part of our team culture [sigh]."
P16	"Our team motto is 'fuel for performance,' so nutrition is ingrained in everything we do."
P4	"There's a sense of camaraderie when we all eat together before games...it brings us closer as a team."
P21	"When the coaching staff emphasizes nutrition, it shows they care about us as athletes, not just players."
P12	"We have team rules about nutrition, like no junk food in the locker room...it helps keep us focused."
P28	"Our team captain is really into nutrition, so he's always sharing tips and recipes with the rest of us."
P14	"The team environment can be competitive, even when it comes to nutrition...we push each other to eat better."
P7	"Our coaches bring in nutrition experts to talk to us, which shows they're invested in our health and performance."
P25	"It's easier to stick to my nutrition plan when my teammates are supportive and encouraging."
P3	"We have team goals for nutrition and hydration, and we track our progress together."
P27	"When we see other teams prioritizing nutrition, it motivates us to do the same [laugh]."
P9	"We have team-building activities centered around cooking and meal prep...it helps us bond and learn about nutrition together."
P20	"Our team culture is all about supporting each other, so we're always looking out for each other's nutrition."
**Theme 5: Suggestions for Improvement – Participants provided valuable insights and recommendations for enhancing the effectiveness of plans.**	

Participant	Specific Transcript Excerpt
P15	"I think having more one-on-one sessions with a nutritionist would be really helpful...they could tailor the advice to each athlete's needs."
P18	"It would be great if we had access to more nutritious options in the cafeteria and at team events [sigh]."
P10	"I'd like to see more educational workshops or seminars on nutrition and its impact on athletic performance."
P26	"We should have nutrition resources available online, so we can access information whenever we need it."
P29	"Having cooking classes or meal prep [laugh]… sessions with a chef would teach us practical skills for eating healthier."
P1	"If the nutrition plan was more flexible and adaptable to our schedules, it would be easier to follow."
P24	"I think setting specific nutrition goals for the team would give us something to work towards together."
P6	"It would help if our coaches provided more guidance on what to eat before and after workouts and games."
P13	"We need more support and encouragement from the coaching staff to stick to our nutrition plans."
P2	"Having a designated nutrition area in the locker room stocked with healthy snacks would be convenient."
P30	"I'd like to see more emphasis on nutrition in our… team meetings and practices...it's often overlooked."
P8	"If we had regular check-ins with a nutritionist, it would help us stay on track and make adjustments as needed."
P19	"We should have team challenges focused on nutrition, like who can try the most new fruits and vegetables in a week."

P17	"[laugh]Offering incentives for reaching nutrition goals, like gift cards or gear, would motivate us to stick to the plan."
P23	"I think having a nutrition app specifically for our team would make it easier to track our food intake and progress [sigh]."
P5	"We should have nutrition education sessions during preseason training to set the tone for the season."
P22	"If the team culture… embraced nutrition more, it would make it easier for everyone to prioritize it."
P16	"Having a team meal plan tailored to our dietary needs and preferences would save us time and stress."
P4	"It would be helpful to have a designated team nutritionist who's available to answer questions and provide guidance."
P21	"Incorporating nutrition education into our academic classes would reinforce its importance and make it more accessible."
